# Non-functioning Pituitary Macroadenoma Resection in Twin Pregnancy: A Case Report

**DOI:** 10.7759/cureus.42891

**Published:** 2023-08-03

**Authors:** Anup Bista, Dipesh Mishra, Suson Ghimire, Prakriti Bhandari, Sudan Puri

**Affiliations:** 1 Anesthesia and Critical Care, Patan Academy of Health Sciences, Kathmandu, NPL; 2 Intensive Care Unit, Chirayu National Hospital and Medical Institute, Kathmandu, NPL; 3 Anesthesia, Patan Academy of Health Sciences, Kathmandu, NPL; 4 Obstetrics and Gynaecology, Pyuthan Hospital, Pyuthan, NPL

**Keywords:** twins, surgery, pregnant woman, pituitary adenoma, anesthesia

## Abstract

Non-functioning pituitary adenomas (NFPAs) are rare in females of reproductive age. We present the case of a 37-year-old pregnant woman in her second trimester with a diagnosis of symptomatic pituitary macroadenoma with twin pregnancy. Magnetic resonance imaging (MRI) confirmed a well-defined macroadenoma compressing the optic chiasm, thus necessitating surgery. The patient underwent transnasal transsphenoidal resection of the tumor under general anesthesia. Anesthesia management posed challenges due to concurrent considerations of pregnancy and neurosurgery. Induction, maintenance, monitoring, and fluid management were carefully performed. The patient experienced a transient decrease in oxygen saturation, which improved with lung recruitment maneuvers. The surgery was successful without any complications. Postoperatively, an ophthalmology consultation was done, which showed an improvement in the patient's vision, as evidenced by the perimetry findings. Later, she delivered healthy twins at 36 weeks and six days of gestation. This case highlights the importance of a multidisciplinary approach and meticulous anesthetic management when dealing with pregnant patients undergoing non-obstetric surgery, ensuring optimal maternal-fetal perfusion and minimizing risks to both the mother and the fetus.

## Introduction

Pituitary adenomas represent approximately 10-15% of all intracranial tumors. Among them, nonfunctioning pituitary adenomas (NFPAs) account for 15-30% of all pituitary adenomas. These NFPAs are rare during reproductive age, with their peak occurrence reported between 40 and 80 years [1]. In women with pituitary adenomas, pregnancy is rare; this may be related to the hormone excess from secretory adenomas, such as prolactinomas or corticotroph adenomas. In healthy women, pituitary gland size increases during pregnancy. Since some pituitary adenomas also enlarge during pregnancy, there is a risk of visual impairment and pituitary apoplexy [2], especially in women with macroadenomas or tumors near the optic chiasm. During pregnancy, surgery is the preferred option for reducing adenoma volume to protect visual function, with cabergoline therapy as an alternative. If surgery is not possible or in the case of non-pregnant patients, radiation therapy can be used to prevent tumor regrowth. Transnasal transsphenoidal (TNTS) pituitary surgery is the preferred approach, providing rapid and midline access to the sella with minimal risks and complications. However, when surgery is necessary, anesthesiologists face the challenge of managing the unique demands of both pregnancy and neurosurgery. This article presents the case of a 37-year-old pregnant woman in her second trimester who underwent TNTS resection of a pituitary tumor, highlighting the complexities and considerations involved in managing such cases.

## Case presentation

**Figure 1:**
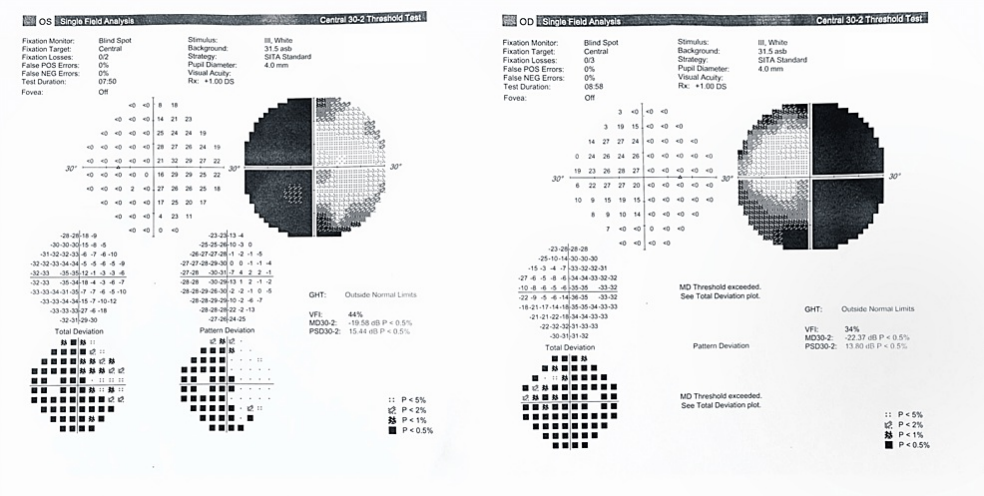
Preoperative perimetry showing bitemporal hemianopia Automated visual field study using the 30-2 threshold test and Swedish Interactive Threshold Algorithm (SITA) standard strategy revealed bitemporal homonymous hemianopia with a visual field index of 44% in the right eye, and a pattern standard deviation (PSD) of 15.44 in the right eye. In the left eye, the visual field index is 34% with a PSD of 13.80.

A 37-years-old parturient (gravida 4, para 1, living 1, abortion 2) with a twin pregnancy at 25 weeks and five days of gestation presented with a complaint of blurred vision that had been ongoing for a year, with worsening symptoms over the past month, which were not present during her initial stages of pregnancy in her antenatal visit. She reported difficulty seeing laterally, but without any associated eye pain. Additionally, she had been experiencing headaches for the past month. She denied other complaints such as vomiting, nipple discharge, or intolerance to heat or cold. Apart from receiving treatment for subfertility for a year prior to conceiving through in vitro fertilization, her medical history was unremarkable. Her ophthalmic examination revealed bitemporal hemianopia (Figure 1).

**Figure 2:**
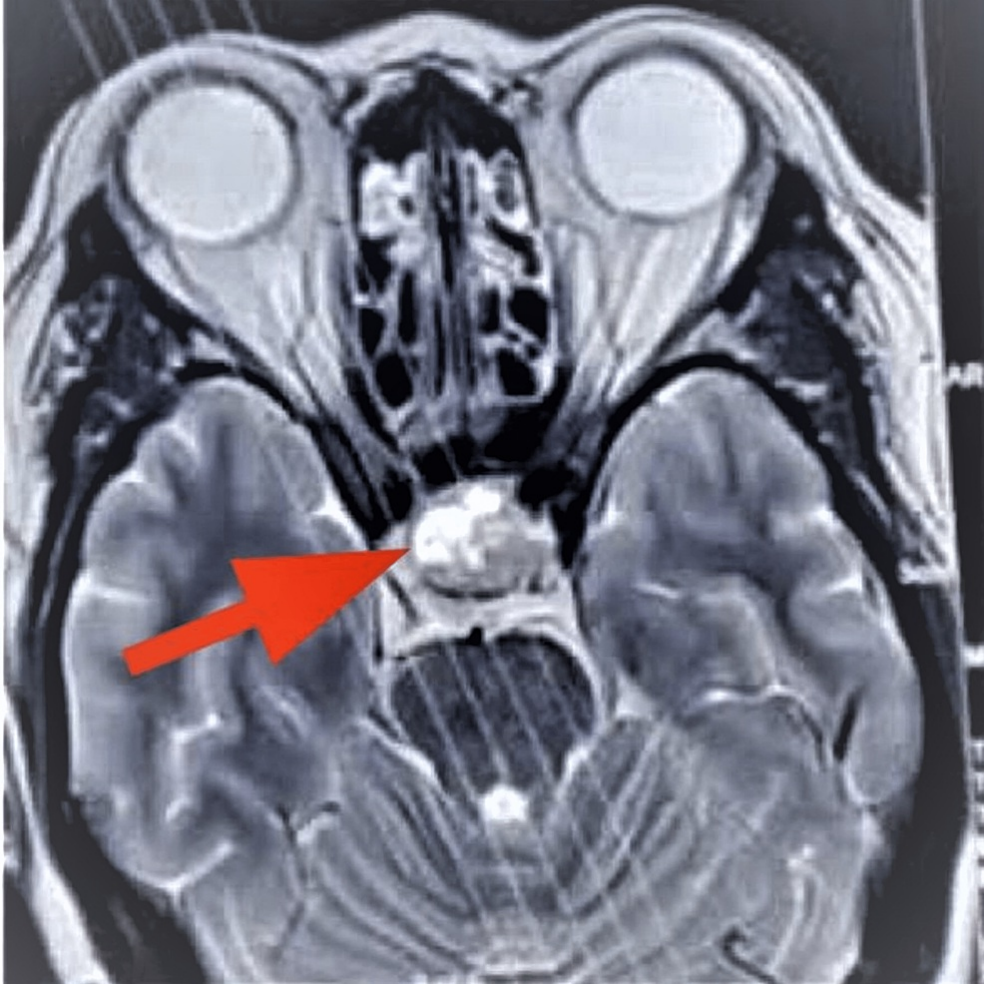
MRI of brain showing pituitary mass

Her MRI revealed a pituitary macroadenoma with apoplexy. The lesion was well-defined, measuring 29mm x 24mm x 25mm and located in the sella and suprasellar regions. There was mild widening of the sella and slight compression of the optic chiasm. Additionally, the tumor was in close proximity to the cavernous part of the left internal carotid artery (Figure 2).

A multidisciplinary team involving neurosurgery, otorhinolaryngology, obstetrics, and anesthesiology collaborated to carefully assess the situation and collectively decided to proceed with surgery. The primary goal of the surgery was to prevent complete blindness in both eyes, considering the potential growth of the tumor and its compression on the visual pathway. The pre-anesthetic assessment, hormone levels (prolactin: 123.44 ng/ml, thyroid stimulating hormone (TSH): 4.39 mIU/L, growth hormone: 0.24 ng/ml, and cortisol: 9.16 mcg/dl), fetal ultrasound, and obstetric scan were all normal. The planned surgical approach was endoscopic endonasal transsphenoidal resection. The patient was positioned in the left lateral position to minimize aortocaval compression. 

Following preoxygenation, anesthesia was induced with intravenous administration of midazolam 2mg, fentanyl 100mcg, propofol 120mg, and rocuronium 50mg. Subsequently, the patient's airway was secured through oral intubation. Anesthesia was maintained using isoflurane, and intermittent doses of vecuronium were administered to sustain neuromuscular blockade throughout the procedure. There was a decrease in saturation of peripheral oxygen (SpO2) from 90% on room air before induction to 88% even with fraction of inspired oxygen (FiO2) being 100%. Lung recruitment maneuvers were performed, resulting in an increase in SpO2 to 95%. Intraoperatively, the patient's heart rate ranged from 90 to 120 beats per minute, the mean arterial pressure from 70 to 100 mm Hg, and the end-tidal carbon dioxide (CO2) from 30 to 35mm Hg. The surgeon used an endoscope to access the sphenoidal sinus through the nasal passage. Subsequently, the thin bone of the sella was carefully removed to expose the dura, allowing for further access to the tumor. The surgical procedure resulted in an estimated blood loss of approximately 150 mL. Following the completion of the two-hour surgery, the patient was transferred to the surgical ICU for staged extubation. 

In the surgical ICU, the patient's vital signs were closely monitored. As a rescue analgesic, a 25mcg fentanyl injection was administered. The patient received continuous positive airway pressure (CPAP) with positive end-expiratory pressure (PEEP) of 5 cmH2O. After a comprehensive clinical assessment, the patient was extubated the same evening, four hours after the surgery was completed.

**Figure 3:**
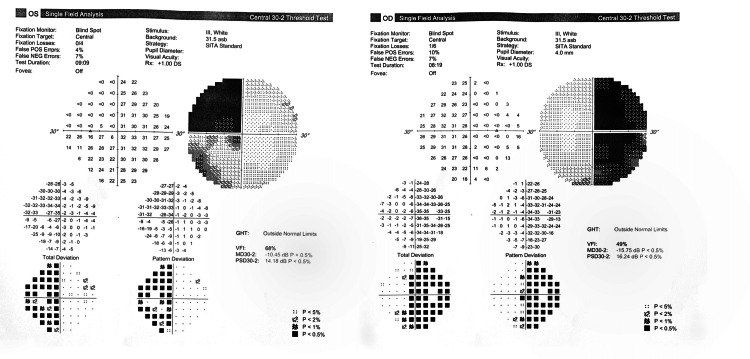
Postoperative perimetry showing an improvement in the visual field Automated visual field study with the central 30-2 threshold test using Swedish Interactive Threshold Algorithm (SITA) standard strategy and stimulus size III in white showed bitemporal hemianopia. The postoperative visual field studies revealed a slight improvement in the field defect. The mean deviation improved from -22.37 dB to -15.75 dB in the right eye and from -19.58 dB to -10.58 dB in the left eye (with p < 0.05%).

During the postoperative stay, pain was managed using a multimodal approach, which included the administration of intravenous paracetamol 1gm every six hours, ketorolac 30mg every 12 hours, and fentanyl 25mcg as needed for additional pain relief. Fortunately, there were no complications during postoperative recovery, such as bleeding, infection, CSF leak, or diabetes insipidus. The patient experienced an improvement in her vision, which was further confirmed by perimetry (Figure 3).

The patient was discharged on the seventh day after the surgery. Histopathology revealed that the tumor belongs to a heterogeneous group, classified based on its pituitary cell lineage. The patient successfully delivered two healthy babies via elective lower segment cesarean section at 36 weeks and six days gestation. Additionally, she did not encounter any difficulties with lactation.

## Discussion

NFPAs account for around 30% of pituitary tumors [3]. They don't cause hormonal hypersecretion and are typically diagnosed in individuals in their sixties. NFPAs may be classified into different subtypes based on their structural and immunohistochemical characteristics, such as gonadotroph adenomas, null cell adenomas, and oncocytomas. However, approximately 15% of NFPAs are considered "silent adenomas", which express hormones only detectable by immunocytochemistry without systemic secretion [4]. 

NFPAs can cause various symptoms such as headaches, vision problems, vomiting, and seizures, which are common in patients as the tumor grows. Patients may experience visual loss due to compression of the optic chiasm leading to bitemporal hemianopia. Surgical decompression can help reverse these symptoms in most cases. 

MRI with gadolinium contrast is necessary for the diagnosis of the tumor. It offers a comprehensive evaluation of the pituitary region along with other related structures like the optic chiasm and the neighboring areas in order to evaluate how much invasion the tumor has made [5]. Hormone level measurements including prolactin, TSH, and cortisol should be done for accurate diagnosis. 

A commonly recommended course of action for treating NFPAs involves transsphenoidal surgery as a primary therapy option [6]. Notwithstanding whether surgical reduction results in full tumor resection, most NFPA patients note some positive outcomes such as improvements with visual field defects, the majority (80%) having significant progress with almost half (40%) achieving complete normalization [7].

When an obstetric patient requires non-obstetric surgery, several anesthesia considerations should be kept in mind. The physiological changes during pregnancy should be taken into account while administering anesthesia. Prenatal consultation and preoperative preparation are important to clarify any issues and provide information about the procedure and anesthesia. Aspiration prophylaxis is crucial and should be provided to all pregnant women during labor if the patient is not fully nil per oral. However, in our case, we made the decision not to administer aspiration prophylaxis as the patient was not in labor, and her oral intake status was well-maintained. Additionally, there is an increased risk of thrombosis during pregnancy, and there is also a concern about bleeding during the operation.

Furthermore, there is a risk of teratogenicity when administering anesthesia during pregnancy. However, in our case, we took precautions by inducing the surgery with propofol and maintaining it with isoflurane, both of which have not been proven to be teratogenic in the fetus to date. The potential of anesthetic pills to be teratogenic is based on their effects on cell signaling, mitosis, and DNA synthesis [8]. However, the evidence of modern clinical practice does not provide an excellent indicator of the teratogenic effects of anesthetics. None of the unique anesthetics are significantly harmful to the fetus [9].

It is always important to maintain uteroplacental perfusion pressure to avoid fetal asphyxia, as the fetus lacks an autoregulatory mechanism. Proper left lateral tilt or wedging should be maintained to avoid aortocaval compression. It is crucial to maintain adequate maternal blood pressure and avoid conditions that can affect blood flow, such as hypovolemia, hypercapnia, severe anemia, maternal hypoxia, and uterine hypertonia. The rate of fetal loss after surgical intervention was 5.80% throughout pregnancy. When considering only first-trimester abortions after surgery, the rate rose to 10.50% [10].

Complications during the surgical treatment of NFPAs are extremely rare. Exceptions include ocular trauma (0.50-2.40%), CSF leakage (1.50-4.20%), carotid artery injury (0.40-1.40%), meningitis (0.50-1.90%), eye disease (0.40-1.90%), and hemorrhage in the remaining tumor tissue (0.80-2.80%) [2]. Temporary diabetes insipidus may occur in up to 15% of surgical cases, but permanent diabetes insipidus is rare, affecting 0.90-5% of patients. The risk of perioperative mortality is low, ranging from 0.20% to 1.20%.

Postoperative pain management is important for maternal comfort. Short-term drug use is generally considered safe, although it is important to note that some medications can cross the placental barrier. However, after 32 weeks of gestation, it is advisable to avoid long-term use of non-steroidal anti-inflammatory drugs (NSAIDs), particularly ibuprofen, due to the potential risk of premature closure of the ductus arteriosus.

## Conclusions

Surgical intervention becomes essential in pituitary adenoma, where severe symptoms like apoplexy and significant visual impairment manifest. Transsphenoidal surgery has become the preferred option over transcranial or transethmoidal routes for addressing these intricate scenarios. This approach offers several advantages, including easy surgical access, reduced risks of bleeding and brain injury, and minimized postoperative complications. Progress in surgical techniques and diagnostic imaging modalities has played a role in enhancing outcomes in pituitary surgery. The successful management of such cases necessitates a multidisciplinary team approach, ensuring comprehensive care for both the mother and the fetus. Conducting further research is crucial for investigating optimal surgical techniques and their potential impact on maternal and fetal complications.
